# No Increase in Response Rate by Adding a Web Response Option to a Postal Population Survey: A Randomized Trial

**DOI:** 10.2196/jmir.9.5.e40

**Published:** 2007-12-31

**Authors:** Jan Brøgger, Wenche Nystad, Inger Cappelen, Per Bakke

**Affiliations:** ^4^Section for NeurologyInstitute of Clinical MedicineUniversity of BergenBergenNorway; ^3^Department of Thoracic MedicineInstitute of MedicineUniversity of BergenBergenNorway; ^2^Norwegian Institute of Public HealthOsloNorway; ^1^Department of NeurologyHaukeland University HospitalHelse Bergen Health AuthorityBergenNorway

**Keywords:** Internet, randomized control trial, questionnaire, epidemiology, response rate, bias

## Abstract

**Background:**

There is substantial interest in use of the Internet for surveys, but there have been few health-oriented, large, randomized trials of general population surveys on the Internet. It is unclear whether providing the option to respond via Internet increases the response rate, and to what degree the results will differ.

**Objective:**

The aim of the study was to evaluate changes in response rate and outcomes in a postal respiratory health survey by adding an optional Web response alternative.

**Methods:**

This was a randomized trial of a random sample of 4213 permanent residents of Norway, aged 20-40 years. Participants were randomized into a traditional survey arm, where they were asked to return the survey by mail, and an arm where they were also offered the option to respond via a Web form.

**Results:**

A total of 1928/4213 subjects responded, a response rate of 45.8% across both arms. The total response rate was 44.8% (944/2105) in the postal plus optional Internet response arm and 46.7% (984/2108) in the usual postal survey arm, with no statistically significant difference between the randomized groups (*P* = .24). In the optional Internet arm, 8.3% (175/2105) of the sample responded using the Internet and 36.5% (769/2105) responded by post. Thus, Internet response was chosen by 18.5% (175/944) of those who replied in the optional Internet arm. In the multivariate analysis, Internet response was associated with being male, frequency and type of Internet access (home users more likely to respond by Internet than work users), and smoking habit, with current smokers being more likely to be Internet responders. 57% preferred postal response (1102/1928), 38% preferred Internet response (733/1928), and 3% preferred telephone interview (54/1928), with no difference between randomization arms (*P* = .56). But among those who indicated that they preferred the Internet response and who were randomized to the optional Internet arm, only 47% actually chose the Internet response. Asthma prevalence was higher among participants choosing the Internet response mode (16.7% vs 12.4%).

**Conclusions:**

We failed to increase survey response rates by adding an optional Internet response. Asthma diagnosis was higher in the Internet response group, suggesting nonresponse bias. Method comparison studies should be carried out before Internet studies are accepted in new populations or new subject matters.

## Introduction

The population survey remains a cornerstone of public health research and epidemiological inference. There is a substantial interest in using the Internet for surveys because of speed, low cost of data collection, and potentially large sample sizes and also because of falling response rates with conventional surveys [1-3]. On the one hand, there is substantial literature on Internet-focused surveys, based on Internet-only populations [4]. An ongoing concern is the possibility of generalizing from these populations accessible through the Internet to general populations. There is both theoretical and empirical evidence of self-selection bias [5,6]. On the other hand, a somewhat separate branch in the rich literature on survey methods has studied ways of using the Internet in a more traditional population survey process [7]. A number of studies have been done in various special populations: students [8,9], businesses, occupational and election surveys, and email lists [10-13]. There is also an academic online database focusing on Internet survey methodology.

However, there are few directly comparative studies that can directly answer the question of what the role of the Internet in a general population survey could be. Such studies would use a geographically defined general population and rigorous randomization to isolate the Internet factor only.

### The Choice of Survey Method

The choice of basic survey method is not always clear cut and varies regionally and by the traditions in the subject field. Large surveys still use in-person interviews (eg, National Health and Nutrition Examination Survey), postal questionnaires (eg, American Community Survey, the successor to the US Census Bureau decennial census), or an eclectic mix of all modes (eg, World Health Organization’s World Health Survey [14]). This growing role of mixed-mode surveys adds additional complexity, as recently reviewed by de Leeuw [15]. For the next few years, the Internet is going to be an optional add-on to more conventional survey methods in general population surveys.

### Scarcity of Randomized Trials

Through various literature searches and contact with experienced survey technologists through survey mailing lists, we were able to find only four prior studies that evaluated use of the Internet for surveying a geographically defined general population in a randomized fashion [16-19].

Norway has an excellent sampling frame available to researchers through the National Central Population Registry. All permanent residents are required by law to register. Internet access is common in Norway, with 42% of Norwegians accessing the Internet daily in 2003 [20]. Thus, Norway is a good setting in which to test whether response rates are increased by adding an Internet response option to a postal survey.

In this study, we performed a randomized trial in adult Norwegians, comparing a regular postal survey with a postal survey that had an optional Internet-based response. Our hypothesis was that the addition of the Internet response option to a postal survey would increase response rate with little bias from the mix of survey modes.

## Methods

### Design

We performed a parallel group randomized trial of the Norwegian general population. In April 2004, we randomly selected 4213 persons aged 20-40 years from the Norwegian Central Population Registry, covering all of Norway. Age, gender, and county of residence were taken from the registry. All other variables were recorded from the questionnaire. Half of the participants were randomized to the postal plus optional Internet response intervention (n = 2105), the rest to the standard postal survey (n = 2108). Simple randomization was performed without stratification or blocking. Participants were blinded to the randomized nature of the study. The study was powered to have 90% power to detect a 5% difference in response rates at an alpha level of .05 (50% vs 55%). The survey was performed from April to August 2004.

### Postal Component

All participants were mailed a one-page introductory letter explaining that the purpose of the study was to establish the occurrence of and risk factors for asthma and allergies in Norway. They were also mailed a one-page questionnaire containing 40 questions on respiratory symptoms and diagnoses that has been used extensively in previous work [21,22], as well as questions on morbidity, known and suspected risk factors, and use of modern communication methods. A pre-paid response envelope addressed to the sponsoring institution was enclosed. The intervention group additionally received a one-page sheet describing the optional use of the Internet response, along with a 7-digit user identification and 4-digit password. One reminder containing another copy of the questionnaire and pre-paid response envelope was sent by mail to nonresponders after 6 weeks.

The postal survey cost €2 (US $1.75) per person in printing and mailing costs, excluding workload. The additional cost of printing and mailing the Internet response explanation sheet and setting up the Web server was approximately €0.35 (US $0.26) per person. Setting up the server took a few days of work for the first author.

### Web Server

The Internet Web server was set up by the first author. The server was a standard commercial Windows server with Active Server Pages (.ASP). There were no client-side scripts or cookies. The opening page was brief (105 words) and contained a prominent user identity and password box. There were 11 text-only questionnaire pages, containing 1-14 questions viewable on all platforms with an 800 × 600 pixel screen without scrolling. Sign-in was performed by entering a numeric user ID and numeric password printed on the questionnaire (Multimedia Appendix 1). A pilot study using friends and coworkers (n = 17) showed that the Web questionnaire was easily completed and took less than 15 minutes. Identity was ascertained through log-in. There were no potentially identifying questions or data such as age, gender, or municipality of residence, and Internet protocol (IP) numbers were not logged.

### Statistical Methods

The primary analysis was by intention-to-treat, comparing response rates overall and in various subgroups. Secondary analyses included predictors of choosing the Internet response in the postal plus optional Internet response arm, as well as changes in the main outcomes of the survey (self-reported demographics and prevalence of outcomes) with survey mode. Change in prevalence of symptoms with number of mailings was taken as an indicator of nonresponse bias. Participant preference for response mode was assessed both by asking about preferred response mode (postal, Internet, or telephone) and by contrasting with their actual choice of survey response mode. The question was “If you could choose how to respond to this or a similar survey, what would you choose?” The options were “Receive a call from an interviewer” or “Receive a questionnaire by mail and…,” then either “Send in the completed form by mail” or “Answer on the Internet.”

Age was categorized as 20-24, 25-30, 30-34, and 35-40 years. All other variables were categorical. County of residence was classified into rural or small urban, medium urban, and large urban based on conventional Norwegian cutoff points according to county population size (< 8000, 8000-50000, > 50000). Statistical comparisons used the chi-square test for univariate analysis, whereas multivariate analyses used multiple binomial logistic regression.

The study was recommended by the Regional Committee for Medical Research Ethics in Norway and had the appropriate permission from the Norwegian Data Protection Authority through a simplified procedure with the Norwegian Social Science Data Services.

## Results

### Response Rates

The randomized groups were well matched for gender, age, and municipality size (Table 1). A CONSORT-style [23] flowchart is available in Multimedia Appendix 2.

**Table 1:** Comparison of randomized arms by age, gender, and population density of municipality of residence

	Postal (n = 2108)	Optional Internet (n = 2105)
**% men**	51.8	49.9
**Age (years), mean (SD)**	30.7 (6.07)	30.7 (6.04)
**Residential density (%)**		
Rural or small urban	43.0	43.0
Medium urban	21.6	23.1
Larger urban	35.4	33.9

A total of 45.8% (1928/4213) individuals responded. The total response rate was 44.8% (944/2105) in the postal plus optional Internet response arm and 46.7% (984/2108) in the usual postal survey arm, with no statistically significant difference between the randomized groups (*P *= .24; Table 2). In the optional Internet arm, 8.3% (175/2105) of the sample responded using the Internet and 36.5% (769/2105) responded by post. There was a significantly lower response rate in the optional Internet arm (36.7%) compared with the usual postal arm (44.2%) in the 20-24 age range (*P *= .02).

Response rates were 31.4% (661/2105) and 31.6% (667/2108) after the first letter, and an additional 13.4% (283 additional responses) and 15.0% (317 additional responses) after the reminder letter in the postal plus optional Internet and usual postal arms, respectively (*P *= .28). Response rates were more than 10% higher in women in both treatment arms (*P *< .001). There was no age trend in response in the usual postal arm (*P *= .23), but a significant trend in the postal plus optional Internet arm (*P *< .001), driven by the lower response rate in the 20-24 age group. There was no trend by residential status. The same results were found when analyzing trends in response rates according to initial and reminder letter response (data not shown).

**Table 2:** Effect of intervention on response rate

		Trial Arms					Within Optional Internet Arm			
		Postal (n = 2108)		Optional Internet (n = 2105)		*P*	Postal Response (n = 769)		Internet Response (n = 175)	
		%	No.	%	No.		%	No.	%	No.
**Gender**										
	Male	41.9	457/1091	38.2	402/1051	.09	29.0	305/1051	9	97/1051
	Female	51.8	527/1017	51.4	542/1054	.86	44.0	464/1054	7	78/1054
**Age (years)**										
	20-24	44.2	200/452	36.7	159/433	.02	29.6	128/433	7	31/433
	25-30	45.3	196/433	44.4	206/464	.84	34.1	158/464	10	48/464
	30-34	45.8	242/528	50.6	252/498	.13	42.4	211/498	8	41/498
	35-40	49.8	346/695	46.1	327/710	.17	38.3	272/710	7	55/710
**Residential density**										
	Rural or small urban	44.4	402/906	44.2	400/906	.96	37.2	337/906	7	63/906
	Medium urban	47.6	217/456	44.0	214/486	.30	35.6	173/486	8	41/486
	Larger urban	48.9	365/746	46.3	330/713	.32	36.3	259/713	10	71/713
**Total**		46.7	984/2108	44.8	944/2105	.24	36.5	769/2105	8.3	175/2105

### Expressed Preference and Chosen Response Mode

Internet access and type of Internet access would seem important explanatory factors in this study. Among all respondents from both arms, more than 90% had access to the Internet: 59% both at work and at home (1144/1928), 15% at home only (291/1928), and 17% at work only (321/1928); however, 7% had no Internet access (125/1928).

An evaluation of the predictors of choosing the Internet response option among the respondents in the optional Internet arm is given in Table 3. In the univariate analysis, Internet response was associated with being male, frequency and type of Internet access, and planned education. In the multivariate analysis, Internet response was associated with being male, frequency and type of Internet access, and smoking habit. The strongest predictor of Internet response was Internet use or access type, followed by gender. Interestingly, current smokers were more likely to be Internet responders.

We evaluated which response mode respondents actually chose as well as their expressed opinion on their preferred response mode. Respondents were asked their preferred method of survey response—postal, telephone interview, or Internet; 57% preferred postal response (1102/1928), 38% preferred Internet response (733/1928), and 3% preferred telephone interview (54/1928), with no difference between randomization arms (*P *= .56). But among those who indicated that they preferred the Internet response and who were randomized to the optional Internet arm, only 47% actually chose the Internet response. While 97% (170/175) of actual Internet respondents expressed a preference for Internet response, 26% (193/752 responders to the question) of the postal respondents in the Internet randomization arm also expressed a preference for Internet response—a “false preference.” In multivariate analysis, this preference discrepancy was not associated with age (*P *= .73) but was strongly associated with male gender (*P *< .001), never smoking (*P *= .02), larger urban residence (*P *= .07), and higher educational achievement (*P *< .001).

We evaluated predictors for choosing the Internet response among the 363 persons in the Internet arm who had an expressed Internet response preference in a logistic regression using gender, smoking, age, planned education, residential density, type of Internet access, and intensity of Internet use. The only significant variable was type of Internet access (*P *= .02): in this group, 50% of those with Internet access at home chose the Internet response option, compared to 23% of those with Internet at home and 20% with no Internet access.

**Table 3:** Percent choosing the Internet response among respondents in the postal plus optional Internet arm (n = 944)

		%	Univariate* P *Value	Multivariate OR	95% CI	Multivariate *P *Value
**Total**		18.5 (175/944)				
	Male	24.1	< .001	1 (ref)		.05
	Female	14.4		0.69	0.47-1.00	
**Age (years)**						
	20-24	19.5	.20	1 (ref)		.14
	25-29	23.3		0.98	0.54-1.77	
	30-34	16.3		0.66	0.36-1.22	
	35-40	16.8		0.58	0.31-1.06	
**Residential status**						
	Rural or small urban	15.8	.13	1 (ref)		.07
	Medium urban	19.2		1.62	0.99-2.64	
	Larger urban	21.5		1.55	1.01-2.39	
**Internet use**						
	Daily	27.3	< .001	1 (ref)		< .001
	Weekly	10.2		0.31	0.18-0.51	
	Seldom	3.4		0.10	0.03-0.33	
	No access	1.6		0.05	0.01-0.37	
Internet access^*^						
	At home	23.2	< .001	1 (ref)		< .001
	At work	5.0		0.13	0.06-0.30	
	None	1.5		0.06	0.1-0.47	
**Educational level now^†^**						
	Primary school	18.6	.31	1 (ref)		.40
	High school/vocational	17.9		0.92	0.42-2.03	
	College	16.7		0.79	0.34-1.79	
	University	24.1		1.26	0.53-2.98	
**Planned education**						
	Primary/high school	15.2	.006	1 (ref)		.15
	College	18.4		0.99	0.62-1.58	
	University	25.7		1.50	0.92-2.45	
**Relationship status**						
	Married	17.3	.83	1 (ref)		.70
	Cohabiting	20.1		1.01	0.62-1.66	
	Single	18.5		0.83	0.48-1.41	
	Other	20.0		1.25	0.59-2.62	
**Smoking habit**						
	Never	17.6	.12	1 (ref)		.005
	Former	15.8		0.89	0.57-1.39	
	Current	23.4		2.06	1.26-3.37	

^*^Multivariate odds ratio estimated from a separate model without Internet use intensity included, due to colinearity.

^†^Multivariate odds ratio estimated from a separate model without planned education included, due to colinearity.

### Main Survey Results by Randomization and Survey Mode

Table 4 shows the main survey results by randomization and chosen response mode. Adding an Internet response mode did not change the overall results of the survey, both for demographic variables and health outcome variables, except for asthma diagnosis. Asthma diagnosis was reported more often in the group randomized to the optional Internet response arm, in both the postal and Internet responders.

Internet responders as a group were somewhat different from postal responders. They were much more likely to be male and somewhat more likely to be smokers and have higher educational aspirations (but not achievements). They were also more likely to report phlegm and morning cough.

**Table 4:** Main survey results (demographics, prevalence of symptoms, and diagnoses), according to randomization and chosen response mode in the optional Internet response arm

		Randomized Arm			Optional Internet Arm		
		Optional Internet (n = 944)	Postal (n = 984)	* P *Value	Internet Response (n = 175)	Postal Response (n = 769)	* P *Value
**Demographic variables**							
	Age (years), mean (SD)	31.2 (5.9)	30.9 (6.1)	.26	30.7	31.4	.17
	Gender, % men	43	46		55	40	< .001
	Smoking habit, former	28	28	.99	24	29	.11
	Smoking habit, current	21	21		26	19	
	Educational level now, primary	6	6	.08	7	6	.30
	Educational level now,high school	47	51		46	47	
	Educational level now, college	31	26		28	32	
	Educational level now, university	15	17		20.1	14	
	Educational level planned, primary/high school	42	44	.56	34	44	.006
	Educational level planned, college	33	31		33	33	
	Educational level planned, university	24	25		33	22	
**Symptoms and diagnoses**							
	Morning cough, % yes	18	20	.44	24	17	.03
	Day cough, % yes	13	14	.74	15	13	.30
	Phlegm, % yes	21	23	.39	30	20	.003
	Chronic cough, % yes	12	11	.62	13	12	.63
	Dyspnea grade 1, % yes	18	19	.81	21	17	.23
	Dyspnea grade 2, % yes	14	11	.09	18	13	.07
	Dyspnea grade 3, % yes	3	2	.06	3	3	.71
	Dyspnea grade 4, % yes	2	1	.50	3	1	.05
	Attacks of breathlessness, % yes	15	13	.19	17	15	.43
	Wheezing ever, % yes	25	23	.30	27	25	.44
	Prolonged episodes of cough and phlegm, % yes, once	18	18	.69	21	18	.29
	Prolonged episodes of cough and phlegm, % yes, multiple times	16	17		18	15	
	Hay fever, % yes	27	27	.89	31	27	.29
	Hay fever, % missing	22	21		18	23	
	Eczema, % yes	19	20	.58	15	20	.14
	Eczema, % missing	20	22		24	19	
	Asthma diagnosis, % yes	13	9	.03	17	12	.18
	Asthma diagnosis, % missing	1	1		0	1	

Phlegm and morning cough were reported more often among those who chose the Internet response. We also performed an adjusted analysis of the association between symptoms and diagnoses and chosen response mode in the Internet arm, adjusting for age,gender, and smoking (not shown). The association remained for phlegm after this adjustment (*P *= .02), but not for morning cough.

For most outcomes, there was no difference in the prevalence of outcomes between initial and reminder letters in either treatment group (data not shown). However, there was a trend in the usual postal group for chronic cough and hay fever: chronic cough was 10% (64/657) versus 15% (46/307) for initial and reminder letter responders, respectively (*P *= .02); hay fever was 29.0% (19/659) versus 23% (70/309; *P *= .01).

## Discussion

We added an easily implemented, low-cost, optional Internet response to a general population postal health survey with randomization, a large sample size, and widespread geographic coverage. We took advantage of a cooperating national population with a well-defined sampling frame and widespread Internet access. The main findings were that response rates were unchanged and there were demographic predictors of Internet response. There was also some indication of bias according to traditional measures, such as differences in asthma prevalence between survey modes and between early and late responders, but for a small number of questions.

The results of this trial regarding response rates are probably generalizable to other countries since access to the Internet in Norway is comparable to many European and North American countries [24] and population surveys are widespread. Cost were negligible and workload light.

We identified three previous optional Internet response studies: two saw a 5% to 15% decrease in response rate [16,18], and one saw a 2.5% increase in response rate [17]. Another study randomized subjects to Web-only response, which gave a poor 15% response rate [19].

As with most previous studies, this was not a study of a true “Internet population survey.” The rationale was that an Internet-only response mode was unlikely to achieve adequate response rates. Our results are similar to these previous studies: no meaningful increase in the response rate by optional Internet response, regardless of subject matter. It is unlikely than an Internet-only response option would generate acceptable response rates within the next few years to be feasible for surveys.

Why was the total response rate not increased? One possibility is technical problems with the website. However, the website was pilot tested and had a very simple design, so this is unlikely. Our Internet response option was meant to increase response rates by reducing the response effort compared to pencil-and-paper response. The high expressed preference for Internet response, but lower use, is puzzling and could shed light on this issue. This preference discrepancy was strongly associated with male gender, higher education, not smoking, and urban residence. This could suggest that Internet response is likely to increase in the future, in that current Internet responders are the “early adopters.” On a more negative note, it could also be an appeasement bias, with responders like to identify with the more recent and novel survey technology. Whatever the case may be, it is likely that participant response effort was not the limiting factor in determining response rates. In particular, we note the reduced response rate in the youngest age group. This was not associated with a similar expressed response mode preference among the youngest responders. It may be that it is easier to put the questionnaire aside when a Web option is included.

It is possible that with increasing adoption of the Internet, an Internet survey will be able to increase response rates, but even in the current population with high Internet use it did not. This remains a hope more than a fact. Tried and tested predictors of survey success, such as topic saliency, remain more important than survey technology, even though the Web option was easy and low cost.

It should also be said that a high response rate is not the end-all or be-all of survey methods. The traditional comparison of late versus early responders did suggest nonresponse bias, but only in the postal group. This traditional indicator was not present in the Internet survey, which is somewhat reassuring. The optional Internet step could even introduce more nonresponse bias if the additional responders were quite different from the target population. On the whole, the only survey result that was affected in the randomized comparison was asthma diagnosis. This was due to a tendency for both postal and Internet responders in the optional Internet arm to respond positively compared to the usual postal arm. Even though it was the only affected outcome, it is still worrying. The asthma diagnosis is a central outcome for the purposes of this survey, and 4 percentage points change in estimated prevalence is substantial. This could be due to failure of randomization, but this is unlikely given the large sample size and good comparability on baseline demographics. It is also unlikely to be a survey mode effect since it was also present in the postal response. One explanation, which we think is likely, is that the optional Internet arm recruited a somewhat different mix of responders. Though response rates were identical, we think that some healthy subjects were put off by the Internet response option, while some persons with asthma, who otherwise would not respond, were particularly attracted to the Internet option.

This study cannot disentangle survey mode effects from nonresponse bias. But looking at who chose the Internet response and how they differ from postal responders could still be instructive. Internet response was associated with some background variables: Internet access, being male, smoking, and educational aspirations. This partly explains the association between two of the symptoms and Internet response. After adjustment for age, gender, and smoking, this association persisted for phlegm. This might be due to residual confounding by smoking intensity or other unmeasured variables. Yet this underscores the potential for unwanted and unexpected survey mode effects and that Internet response options should not be added to a survey naively.

In conclusion, there was no gain in total response rate by adding an Internet response option to a traditional postal questionnaire survey. Adding an Internet-based response option is feasible and low cost. Asthma diagnosis was higher in the Internet arm, suggesting nonresponse bias. Method comparison studies should be carried out before Internet studies are accepted in new populations or new subject matters.

## Multimedia Appendix 1

Complete website files (containing Active Server Pages code and a Microsoft Access database)
									

## Multimedia Appendix 2

CONSORT-style flowchart and checklist
									
