# Supplementary material for: No Increase in Response Rate by Adding a Web Response Option to a Postal Population Survey: A Randomized Trial
Source: J Med Internet Res. 2007 Dec 31;9(5):e40. doi: 10.2196/jmir.9.5.e40 (PMC2270416; doi:10.2196/jmir.9.5.e40)
Supplement: Supplementary file 1 [file jmir_v9i5e40_app1.zip › innhold20040302/deltakerinformasjon.asp]

Deltakerinformasjon


# Undersøkelse om astma og allergi

## Forespørsel om deltagelse i forskningsprosjekt

Ved Nasjonalt Folkehelseinstitutt og Universitetet i Bergen utfører vi nå en
undersøkelse over astma, allergi og røykevaner i Norge. Vi har trukket et
tilfeldig utvalg på 3 000 personer fra Folkeregisteret som blir bedt om å svare
på et spørreskjema, og du er en av disse.

Prosjektet er finansiert av Norsk Folkehelseinstitutt og Universitetet i
Bergen. Resultatene fra undersøkelsen vil bli presentert i media og i
fagtidsskrifter. Det er frivillig å svare, men det er av største betydning for
undersøkelsen at flest mulig fyller ut skjemaet.

Vi ber deg om å svare innen 14 dager. Selv om du velger å delta, kan du fritt
trekke deg på et hvilket som helst tidspunkt og få allerede innsamlede
opplysninger slettet.

Undersøkelsen er meldt til Datatilsynet. Skjemaene er nummerert fordi vi vil
sende ut en påminnelse til de som ikke har svart på dette brevet. Alle
opplysninger vil bli behandlet fortrolig. De som arbeider med prosjektet har
taushetsplikt både som forskere og helsepersonell. Dette prosjektet er ferdig i
år 2009, og opplysningene vil bli anonymisert da. Skulle du ha noen spørsmål om
prosjektet eller spørreskjemaet, ta gjerne kontakt med undertegnede på telefon:
92867303.

Wenche Nystad, seniorforsker, Nasjonalt Folkehelseinstitutt

Jan Brøgger, stipendiat, Universitetet i Bergen

Per Bakke, overlege, professor, Haukeland sykehus, Universitetet i Bergen

Tilbake til startsiden
