# Supplementary material for: No Increase in Response Rate by Adding a Web Response Option to a Postal Population Survey: A Randomized Trial
Source: J Med Internet Res. 2007 Dec 31;9(5):e40. doi: 10.2196/jmir.9.5.e40 (PMC2270416; doi:10.2196/jmir.9.5.e40)
Supplement: Supplementary file 1 [file jmir_v9i5e40_app1.zip › innhold20040302/feil.asp]

Det ble en feil i spørreskjema


# Det ble en feil i spørreskjema.

# Trykk her for å forsøke på nytt.

Eventuelt kunne du være så vennlig å gi beskjed til administratoren for
nettstedet ? Klikk her for å sende epost
til administratoren.

Eller trykk her for å komme til Nasjonalt
Folkehelseinstitutt sine vevsider.
