# Supplementary material for: No Increase in Response Rate by Adding a Web Response Option to a Postal Population Survey: A Randomized Trial
Source: J Med Internet Res. 2007 Dec 31;9(5):e40. doi: 10.2196/jmir.9.5.e40 (PMC2270416; doi:10.2196/jmir.9.5.e40)
Supplement: Supplementary file 1 [file jmir_v9i5e40_app1.zip › innhold20040302/logonvalid.asp]

New Page 1
<%
'Modified from:
'http://www.frontpagehowto.com/newpassword/default.asp
'Userid table: valid. Username: brukernavn. Password: pin
'First we create a connection object
Set Conn = Server.CreateObject("ADODB.Connection")
'Next, we open the connection object by calling the connection string
'that FrontPage created and stored in the global.asa file when the "store"
'connection was created
Conn.Open Application("valid\_ConnectionString")
'Then we create a record set object and a SQL statement
Set RS = Conn.Execute ("SELECT \* From bruker WHERE brukernavn = '" & Request.Form("brukernavn") & "' AND pin = '" & Request.Form("pin") & "'")
'Loop through the database to check for the users information
Do until RS.EOF
pin = RS("pin")
brukernavn = RS("brukernavn")
RS.MoveNext
Loop
'Close the recordset and database connection
RS.Close
Conn.Close
'If the password given is not in the database then we don't do anything, only create an error message.
'Otherwise, we create the session objects
If pin <> "" Then
Session("pin") = pin
Session("brukernavn") = brukernavn
'Redirect everybody with a valid identification to the first page of the questionnaire
Response.redirect("svarside\_1luftvei.asp")
End IF
%>

<% If pin <> "" Then %>
Vent litt, du blir videresendt til sp�rreskjemaet...
<% Else %>
Brukernavnet og PIN-koden matchet ikke, eller finnes ikke i databasen.
  
Klikk her for � komme tilbake til starten
<% End IF %>
