# Supplementary material for: No Increase in Response Rate by Adding a Web Response Option to a Postal Population Survey: A Randomized Trial
Source: J Med Internet Res. 2007 Dec 31;9(5):e40. doi: 10.2196/jmir.9.5.e40 (PMC2270416; doi:10.2196/jmir.9.5.e40)
Supplement: Supplementary file 1 [file jmir_v9i5e40_app1.zip › innhold20040302/takk.asp]

Tusen takk for at du deltok


# Du er ferdig.Tusen takk for at du deltok!

Du kan lukke dette vinduet n�.

Eller trykk her for � komme til Nasjonalt
Folkehelseinstitutt sine vevsider.
