# Supplementary figures and images for: No Increase in Response Rate by Adding a Web Response Option to a Postal Population Survey: A Randomized Trial
Source: J Med Internet Res. 2007 Dec 31;9(5):e40. doi: 10.2196/jmir.9.5.e40 (PMC2270416; doi:10.2196/jmir.9.5.e40)

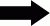

Supplement: Supplementary file 1 [file jmir_v9i5e40_app1.zip › innhold20040302/images/arrow2.gif]

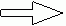

Supplement: Supplementary file 1 [file jmir_v9i5e40_app1.zip › innhold20040302/images/arrow3.gif]

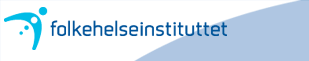

Supplement: Supplementary file 1 [file jmir_v9i5e40_app1.zip › innhold20040302/images/fhi_logo.gif]

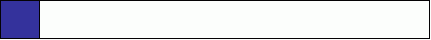

Supplement: Supplementary file 1 [file jmir_v9i5e40_app1.zip › innhold20040302/images/linjal1.gif]

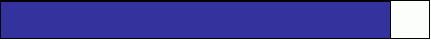

Supplement: Supplementary file 1 [file jmir_v9i5e40_app1.zip › innhold20040302/images/linjal10.gif]

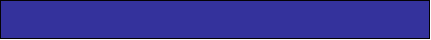

Supplement: Supplementary file 1 [file jmir_v9i5e40_app1.zip › innhold20040302/images/linjal11.gif]

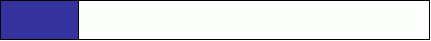

Supplement: Supplementary file 1 [file jmir_v9i5e40_app1.zip › innhold20040302/images/linjal2.gif]

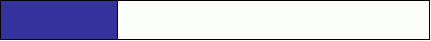

Supplement: Supplementary file 1 [file jmir_v9i5e40_app1.zip › innhold20040302/images/linjal3.gif]

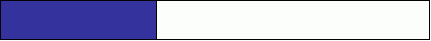

Supplement: Supplementary file 1 [file jmir_v9i5e40_app1.zip › innhold20040302/images/linjal4.gif]

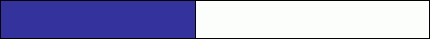

Supplement: Supplementary file 1 [file jmir_v9i5e40_app1.zip › innhold20040302/images/linjal5.gif]

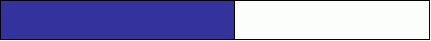

Supplement: Supplementary file 1 [file jmir_v9i5e40_app1.zip › innhold20040302/images/linjal6.gif]

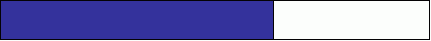

Supplement: Supplementary file 1 [file jmir_v9i5e40_app1.zip › innhold20040302/images/linjal7.gif]

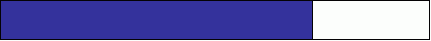

Supplement: Supplementary file 1 [file jmir_v9i5e40_app1.zip › innhold20040302/images/linjal8.gif]

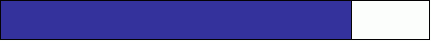

Supplement: Supplementary file 1 [file jmir_v9i5e40_app1.zip › innhold20040302/images/linjal9.gif]

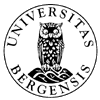

Supplement: Supplementary file 1 [file jmir_v9i5e40_app1.zip › innhold20040302/images/ugle1.gif]
